# Lifetime extension of humpback whale skin fibroblasts and their response to lipopolysaccharide (LPS) and a mixture of polychlorinated biphenyls (Aroclor)

**DOI:** 10.1007/s10565-018-09457-1

**Published:** 2019-01-10

**Authors:** Michael Burkard, Susan Bengtson Nash, Gessica Gambaro, Deanne Whitworth, Kristin Schirmer

**Affiliations:** 1 School of Environment and Science, Griffith University, Brisbane, QLD Australia; 2 Swiss Federal Institute of Aquatic Science and Technology (Eawag), Überlandstrasse 133, CH-8600 Dübendorf, Switzerland; 3 School of Veterinary Science, The University of Queensland, Gatton, QLD Australia; 4 Australian Institute for Bioengineering and Nanotechnology, The University of Queensland, St Lucia, QLD Australia; 5 Institute of Biogechemistry and Pollutant Dynamics, ETH Zürich, Zürich, Switzerland; 6 School of Architecture, Civil and Environmental Engineering, EPF Lausanne, Lausanne, Switzerland

**Keywords:** Cell line transfection, Humpback whale, *Megaptera novaeangliae*, Immunotoxicity, Inflammatory cytokines, Relative telomerase activity

## Abstract

Marine mammals, such as whales, have a high proportion of body fat and so are susceptible to the accumulation, and associated detrimental health effects, of lipophilic environmental contaminants. Recently, we developed a wild-type cell line from humpback whale fibroblasts (HuWa). Extensive molecular assessments with mammalian wild-type cells are typically constrained by a finite life span, with cells eventually becoming senescent. Thus, the present work explored the possibility of preventing senescence in the HuWa cell line by transfection with plasmids encoding the simian virus large T antigen (SV40T) or telomerase reverse transcriptase (TERT). No stable expression was achieved upon SV40 transfection. Transfection with TERT, on the other hand, activated the expression of telomerase in HuWa cells. At the time of manuscript preparation, the transfected HuWa cells (HuWa_TERT_) have been stable for at least 59 passages post-transfection. HuWa_TERT_ proliferate rapidly and maintain initial cell characteristics, such as morphology and chromosomal stability. The response of HuWa_TERT_ cells to an immune stimulant (lipopolysaccharide (LPS)) and an immunotoxicant (Aroclor1254) was assessed by measurement of intracellular levels of the pro-inflammatory cytokines interleukin (IL)-6, IL-1β and tumour necrosis factor (TNF)-α. HuWa_TERT_ cells constitutively express IL-6, IL-1β and TNFα. Exposure to neither LPS nor Aroclor1254 had an effect on the levels of these cytokines. Overall, this work supports the diverse applicability of HuWa cell lines in that they display reliable long-term preservation, susceptibility to exogenous gene transfer and enable the study of humpback whale-specific cellular response mechanisms.

**Electronic supplementary material:**

The online version of this article (10.1007/s10565-018-09457-1) contains supplementary material, which is available to authorized users.

## Introduction

Long-lived cetaceans, such as humpback whales (*Megaptera novaeangliae*), are continuously exposed to lipophilic and bioaccumulative environmental contaminants. The migratory and fasting life history behaviour of this species results in seasonal remobilisation of these toxicants (Bengtson Nash et al. 2013). Further, the timing of elevated circulating contaminant levels coincides with early stages of pregnancy and nursing of new-born calves, intensifying associated toxicological risk (Bengtson Nash 2018). To date, there is little information regarding the toxicological sensitivity of the species (Bengtson Nash et al. 2014; Waugh et al. 2011), and no information specifically related to the influence of contaminants on the immune health of humpback whales; studies are limited to postmortem examination (Apprill et al. 2014; Holyoake et al. 2012) precluding controlled experimentation. Thus, suitable approaches for the quantitative evaluation of species-specific toxicological and immune-toxicological risks in cetaceans are urgently needed; in vitro approaches present one option for the elucidation of toxicological hazards and molecular responses to different stressors at the cellular level.

Recently, we established a wild-type cell line from humpback whale skin (HuWa) (Burkard et al. 2015), which we designated HuWa_wild-type_ for the purpose of the current study. The long-term survival of HuWa_wild-type_ is unknown. Mammalian cells generally undergo only a limited number of proliferation cycles (40–60) until cells enter an irreversible state of growth arrest and senescence known as the Hayflick limit (Hayflick 1965; Nakagawa and Opitz 2007). Consequently, various transfection strategies have been developed to introduce foreign DNA into mammalian wild-type cells in order to control the cell cycle and cellular senescence. One commonly applied transfection strategy is expression of the simian virus 40 large T antigen (SV40T), which is expressed upon infection with the polyomavirus SV40. Expression of SV40T antigen inactivates tumour suppressor genes, such as *p53* and the Rb family, and therefore enables continuous mitosis and long-term survival (Ali and DeCaprio 2001; Ahuja et al. 2005; Wright and Shay 1992).

Another approach to overcoming cellular senescence is by preventing telomere shortening (Wright et al. 1989). Telomeres, which are located at the end of eukaryotic chromosomes, contain a series of nucleotide sequences; loss of these leads to inhibition of the cell cycle, activation of tumour suppressor proteins and, ultimately, senescence (Vaziri and Benchimol 1996; Di Leonardo et al. 1994; Sherr and DePinho 2000). Telomeres are synthesised by the ribonucleoprotein telomerase, which consists of an RNA template and the catalytically active telomerase reverse transcriptase (TERT). It has been shown in human fibroblasts that, if TERT expression is activated, cells can be continuously maintained (Bodnar et al. 1998).

Over-expression of the SV40T and TERT target proteins has been performed in a wide range of mammalian cell lines; however, only a few studies report on SV40T-associated long-term cultivation of cetacean-derived cell cultures (e.g. epithelial cells from bottlenose dolphin (*Tursiops truncatus*) (Pine et al. 2004; Yu et al. 2005), humpback dolphin (*Sousa plumbea*) fibroblasts (Jin et al. 2013) and Yangtze finless porpoise (*Neophocaena phocaenoides*) fibroblasts (Wang et al. 2011). No previous study has reported on TERT transfection of cells from a marine mammal.

This study aimed to firstly establish a reliable and consistently available humpback whale cell culture model as an investigative tool for further toxicological and other investigations. Secondly, the study sought to gain knowledge on the potential application of these cells for assessing immune regulation under non-stress conditions and in immune challenged or chemically stressed humpback whale cells.

To this end, HuWa_wild-type_ cells were transfected with plasmids containing SV40T or TERT, and successful clones were characterised regarding their growth, chromosomal stability and sub-cellular structure. Transfected HuWa cells were treated either with lipopolysaccharide (LPS) or Aroclor (polychlorinated biphenyl (PCB) mixture), and the expression of inflammatory cytokines was quantitatively assessed using anti-human antibodies. LPS is a component of gram-negative bacteria and can stimulate physiological immune reactions by expression of cytokines, chemokines and general stress markers (He et al. 2009; Alexander and Rietschel 2001). Aroclor1254 is a commercially produced PCB mixture. PCBs are ubiquitous and problematic environmental contaminants that are frequently detected in humpback whale blubber (Bengtson Nash et al. 2018; Waugh et al. 2014; Bengtson Nash et al. 2013) and which have been shown to trigger immuno-toxic effects in other marine mammals (Desforges et al. 2016; Mori et al. 2008; Levin et al. 2014; Desforges et al. 2017).

## Methods

### Reagents

All chemicals were ordered from Sigma-Aldrich (Buchs, Switzerland), and all cell culture ware, buffers and media from Life Technologies Invitrogen (Basel, Switzerland), unless otherwise stated. Cell culture flasks were purchased from TPP (Transadingen, Switzerland) and cell culture plates from Greiner Bio-One (Frickenhausen, Germany). Mycoplasma testing was performed approximately every 2 months using the MycoAlert detection kit (Lonza, Visp, Switzerland) and was always found to be negative.

### Cell culture and transfection

The HuWa cell line was previously derived from a dermal biopsy of a free swimming male humpback whale (Burkard et al. 2015). Cells were maintained in HuWa medium, i.e. DMEM/F12 medium supplemented with 10% fetal bovine serum (FBS), 0.1 M non-essential amino acids, 1 M sodium pyruvate and 1% of 5.000 U/ml penicillin–streptomycin, at 37 °C and 5% CO_2._ The medium was changed every second day. At 80–90% confluency, cells were passaged at a ratio of 1:3.

#### Plasmid preparation and transfection

Two plasmids containing *SV40T* (pBABE-puro-SV40 LT and pCEP4-hygro-SV40-Tg) and one plasmid containing h*TERT* (pBABE-puro-hTERT) were purchased from Addgene (Cambridge, USA) (Table S1). Plasmids were amplified in *Escherichia coli DH5α* according to standard protocols, and the plasmid DNA was isolated using the Wizard SV Minipreps DNA purification system (Promega, Duebendorf, Switzerland) following the manufacturer’s protocols. For transfection, HuWa1 cells (passages 8–12; Burkard et al. 2015) were plated into 6-well cell culture plates at a density of 2.6 × 10^4^ cells/cm^2^, 48 h prior to transfection. Two transfection reagents were tested: liposome-based Lipofectamine LTX (Thermo Fisher, Reinach, Switzerland) and non-liposomal Fugene HD (Promega, Duebendorf, Switzerland). Transfections were performed following manufacturer’s protocols.

#### Selection and cloning

Following transfection, cells were allowed to recover for 24 h in HuWa medium. Subsequently, they were passaged in 6-well plates and cultured in antibiotic selection medium consisting of HuWa medium supplemented with 100 μg/ml hygromycin (pCEP4-hygro-SV40-Tg) or 25 μg/ml puromycin (pBABE-puro-SV40 LT and pBABE-puro-hTERT) (Fig. S1). Antibiotics were applied at the lowest concentration required to kill 100% of HuWa_wild-type_. Antibiotic-resistant clones were observed from day 10 onwards. Individual clones were isolated using 10-mm cloning cylinders (Sigma, Saint Louis, USA) and expanded as per standard protocols.

#### Immunocytochemistry: SV40T

Immunocytochemical staining was used to verify SV40T expression. SV40T-transfected cells were seeded (1.7 × 10^4^ cells/cm^2^) onto coverslips within 24-well plates. After 48 h of growth, cells were washed with 1× PBS, fixed with 3.7% paraformaldehyde in PBS for 20 min at RT, permeabilised in 0.2% Triton X-100 in PBS for 30 min, blocked with 4% goat serum in PBS and incubated with Image-iT® FX signal enhancer (Molecular Probes, Eugene, USA) for 20 min. Cells were incubated with the primary antibody (mouse monoclonal to SV40T-antigen; Abcam 16879) at a dilution of 5 μg/ml in 1% goat serum/0.05% Triton X-100 in PBS at 4 °C overnight. Subsequently, samples were washed three times with 0.05% Triton X in PBS and incubated with the secondary antibody (Alexa Fluor® 546 Goat Anti-Mouse IgG (H+L)) at a dilution of 1:1000 in 0.05% Triton X in PBS for 1 h at RT. 4′,6-Diamidin-2-phenylindol (DAPI) was used for nuclear staining and applied before mounting under coverslips with ProLong Antifade (Molecular Probes, Eugene, USA). The vimentin cytoskeleton was visualised by simultaneously immunostaining with a rabbit monoclonal antibody to vimentin (Abcam ab92547, 1:500; secondary antibody Alexa Fluor® 488 Goat Anti-Rabbit IgG (H+L), 1:1000).

#### Telomerase activity

Telomerase activity was assessed using TeloTAGGG Telomerase PCR ELISA (Roche, Mannheim, Germany) following the manufacturer’s protocol. In brief, 2 × 10^5^ HuWa_TERT_ cells were harvested and lysed, and telomeric repeat amplification was performed with provided substrate primers (20-min elongation, 5-min inactivation and 30 amplification cycles). Products were denatured and hybridised with digoxigenin (DIG), followed by immobilisation with biotin and streptavidin coating. Samples were semi-quantitatively assessed using the internal standard and horseradish peroxidase (Anti-DIG-HRP), which is sensitive to tetramethylbenzidine (TMB). The limit of detection (LOD) was considered as the twofold background activity. The data for telomerase activity is shown for 10,000 cell equivalents.

#### Growth curve

HuWa_TERT_ cells were seeded into 12-well plates at a density of 8.5 × 10^3^ cells/cm^2^. Cells were counted after 1, 3, 5 and 7 days using an electronic particle counter (Casy TTC, Schaerfe System, Reutlingen, Germany). Population doubling time (PDT) was calculated from the log phase of the growth curve using the following formula: PDT = log2 / (logN_2_ – logN_1_) × *t*, where N_1_ = cell number/ml at day 3 and N_2_ = cell number/ml at day 7.

#### Karyotype

Two clones of HuWa_TERT_ at passage 26 were treated with colcemid (Invitrogen, Basel, Switzerland) and fixed with a solution of 25% acetic acid and 75% methanol as per standard protocols. A minimum of 20 metaphase spreads for each clone were analysed by Cell Guidance Systems (Cambridge, UK) using standard G-banding procedures.

#### Fluorescence imaging of organelles

Cells were seeded onto coverslips at 1.7 × 10^4^ cells/cm^2^. After 48 h, cells were stained with the lipophilic stain Nile Red (AAT Bioquast, Sunnyvale, CA, USA) at a ratio of 1:5000 in culture medium for 20 min at 37 °C. The endoplasmic reticulum was stained with ER-Tracker (Thermo Fisher, Reinach, Switzerland) at a ratio of 1:2000 in medium for 15 min at 37 °C. Nuclei were stained with NucBlue (Thermo Fisher, Reinach, Switzerland) for 15 min at 37 °C. Cells were visualised immediately using scanning confocal microscopy (Leica, Heerbrugg, Switzerland).

#### Scanning electron microscopy

Cells were seeded at 1.7 × 10^4^ cells/cm^2^ on 12-mm carbon-coated coverslips. Cells were washed with PBS and fixed for 1 h at room temperature using 2.5% glutaraldehyde. After three PBS washing steps, cell monolayers were incubated with 1% OsO_4_ for 30 min, washed again with PBS and immersed in 1% thiocarbohydrazide for 30 min. Thereafter, cells were again incubated with 1% OsO_4_ for 30 min and treated with 10% ionic liquid (Hitachi IL-1000) for 5 min followed by short dips in dH_2_O. The coverslips were air dried and mounted onto 12-mm aluminium scanning electron microscopy (SEM) stubs with conductive carbon cement (Leit-C). Imaging was performed top down in a Helios 600i FEI focused ion beam scanning electron microscope by secondary electron detection (ETD) at 2 kV and 0.34 nA. Cross sections were generated by gallium ion milling at 30 kV and 790 pA and performed on a tilted stage which was perpendicular to the cell monolayer. The images were tilt corrected for perspective distortion. SEM was performed by ScopeM (ETH Zürich, Switzerland).

### Immuno-toxicological investigation

All immune stressor experiments were performed in medium containing FBS. Because FBS contains hormones and growth factors which can influence the expression of cytokines in cells, we reduced the influence of these undefined factors by performing experiments in DMEM/F12 supplemented with 1% FBS, rather than the usual 10% FBS. Cells in 1% FBS were around 75% as metabolically active as those in 10% FBS (Fig. S2). Before treatment, cells were seeded at a density of 2.5 × 10^4^ cells/cm^2^ in 12-well plates and cultured for 4 days until they had formed confluent monolayers, after which time they were used in the following analyses.

#### Cell viability assessment

Metabolic activity and membrane integrity were assessed by the fluorescent indicator dyes alamarBlue (AB) and 5-carboxyfluorescein diacetate acetoxymethyl ester (CFDA-AM) as described in Schirmer et al. (1997). Briefly, upon treatment, the medium was discarded, cells were washed once with 1× PBS and incubated for 25 min with 5% (*v*/*v*) AB and 4 μM CFDA-AM in 1× PBS at 37 °C. Fluorescence was measured at excitation/emission wavelengths of 493/541 and 530/595 nm for AB and CFDA-AM, respectively (Tecan, Infinite M200, Maennedorf, Switzerland). The results were expressed as a percentage of the appropriate control, which was set to 100%.

#### Quantification of inflammatory cytokines

The literature was systematically scanned for proteins that are expressed by fibroblasts, and the reaction of selected targets upon treatment with LPS and PCBs was reported (detailed in Table S2). The following cytokines/chemokines were selected in order to evaluate the impact of LPS and Aroclor in HuWa_TERT_: interleukin (IL)-6, IL-1β, C-X-C motif chemokine ligand 10 (CXCL10), heat shock protein 70 (HSP70) and interferon (IFN)-γ.

Prior to analysis, cells were washed twice with 1× PBS (4 °C) and lysed in 200 μl 1× cell lysis buffer (RayBiotech, GA, USA) containing 1× protease inhibitor concentrate. Cells were re-suspended by pipetting on ice, incubated for 30 min at 4 °C, followed by centrifugation (14,000*×g*, 10 min, 4 °C). Cell lysates were stored at − 80 °C until analysis. Before the application of ELISA kits, the protein content was assessed with a bicinchoninic acid (BCA) protein assay kit (Pierce, Rockford, USA) following the instructions of the manufacturer, and added protein contents were adjusted to 1000 μg/ml total protein.

ELISA kits for human IL-6, IL-1β and IFN-γ were purchased from Qiagen (Hombrechtikon, Switzerland), those for human CXCL10 and HSP70 were ordered from Invitrogen (Basel, Switzerland) and for TNFα from RayBiotech (GA, USA). Quantitative assessment of the respective proteins was conducted according to the manufacturer’s protocols. Briefly, cell lysates were incubated overnight at 4 °C with the respective immobilised antibody, followed by incubation with biotinylated antibody and HRP/streptavidin. The absorbance of the complex was assessed at 450 nm using the Tecan fluorescence plate reader. The limit of quantification was 21.4 pg/ml (TNFα), 14.0 pg/ml (IL-6), 16 pg/ml (IL-1β), 2.0 ng/ml (HSP70), 31.3 pg/ml (IFN-γ) and 2.0 pg/ml (CXCL10).

#### Assessment of inflammatory response to chemical exposure

Stimulation experiments were carried out with LPS from *E. coli* 055:B5 (Sigma-Aldrich, Buchs, Switzerland)*.* LPS was dissolved in deionised nano-pure water and applied by direct dosing into respective wells; cell viability was assessed at 0.1, 1, 10, 25, 50 and 100 μg/ml, and for immune stimulation, a non-cytotoxic concentration (ntC) of 10 μg/ml was selected.

For Aroclor exposure, a stock solution of Aroclor (20 mg/ml) was prepared in dimethyl sulfoxide (DMSO). Final nominal concentrations (100, 600, 1250, 2500, 5000, 7500 and 50,000 μg/l) were achieved by adding 32.5 μl of the appropriate Aroclor concentration into 13 ml of exposure medium (DMEM/F12; 1% FBS; 0.25% DMSO) using amber vials. Subsequently, 2 ml of the final Aroclor concentration was added to respective wells of the 12-well plate. To assess the impact of Aroclor on the inflammatory cytokines (as listed in “Quantification of inflammatory cytokines” section), a non-cytotoxic concentration (ntC) of 1700 μg/l was applied. All plates were covered with aluminium foil and cells were exposed at 37 °C with constant shaking at 250 rpm for 24 h. Immune treatment experiments were conducted with HuWa_TERT_ cells of passages 35–42.

#### Data and statistical analysis

The data shown represent the mean of three independent biological replicates with cells from different passages unless indicated otherwise. Error bars represent standard deviation (SD) unless otherwise stated. Statistical differences were assessed for the LPS and Aroclor treatments by comparing the mean of the three independent experiments for each treatment and the unexposed control using a one-way ANOVA with Bonferroni’s post hoc test; *p* < 0.05 was considered as significant. The analysis was done using GraphPad Prism Version 7 (La Jolla, CA, USA). The non-cytotoxic concentration (ntC) of Aroclor was identified as described by Stadnicka-Michalak et al. (2017).

## Results

### Transfection and characterisation of the new strain: HuWa_TERT_

HuWa_wild-type_ cells were transfected with plasmids containing either *SV40T* or h*TERT*. The two tested transfection reagents, Fugene and Lipofectamine, were both suitable to transfect HuWa_wild-type_. After transfection, antibiotic selection was applied with the first resistant cells appearing after ≈ 7 days (Fig. S3). All three plasmids (pBABE-puro-SV40 LT, pBABE-puro-hTERT and pCEP4-hygro-SV40-Tg) were successfully transfected into HuWa_wild-type_ (Table S3).

In order to assess whether each of the SV40T plasmids (pBABE-puro-SV40 LT; pCEP4-hygro-SV40-Tg) had been successfully introduced, and were also functional, the expression of SV40T was assessed in SV40T-transfected clones (HuWa_SV40T_). Nuclear SV40T immunostaining was detected in only a few cell nuclei for each of the plasmids (Fig. S4). Post-transfection, HuWa_SV40T_ clones divided vigorously for 4–5 days, followed by gradually decreasing proliferation rates and a maximum life span of 15 passages, while most clones had ceased to proliferate at earlier passages. Further, cells increased in size and appeared more polygonal, rather than fusiform, in shape. This behaviour was observed for both SV40T containing plasmids.

**Fig. 1:**
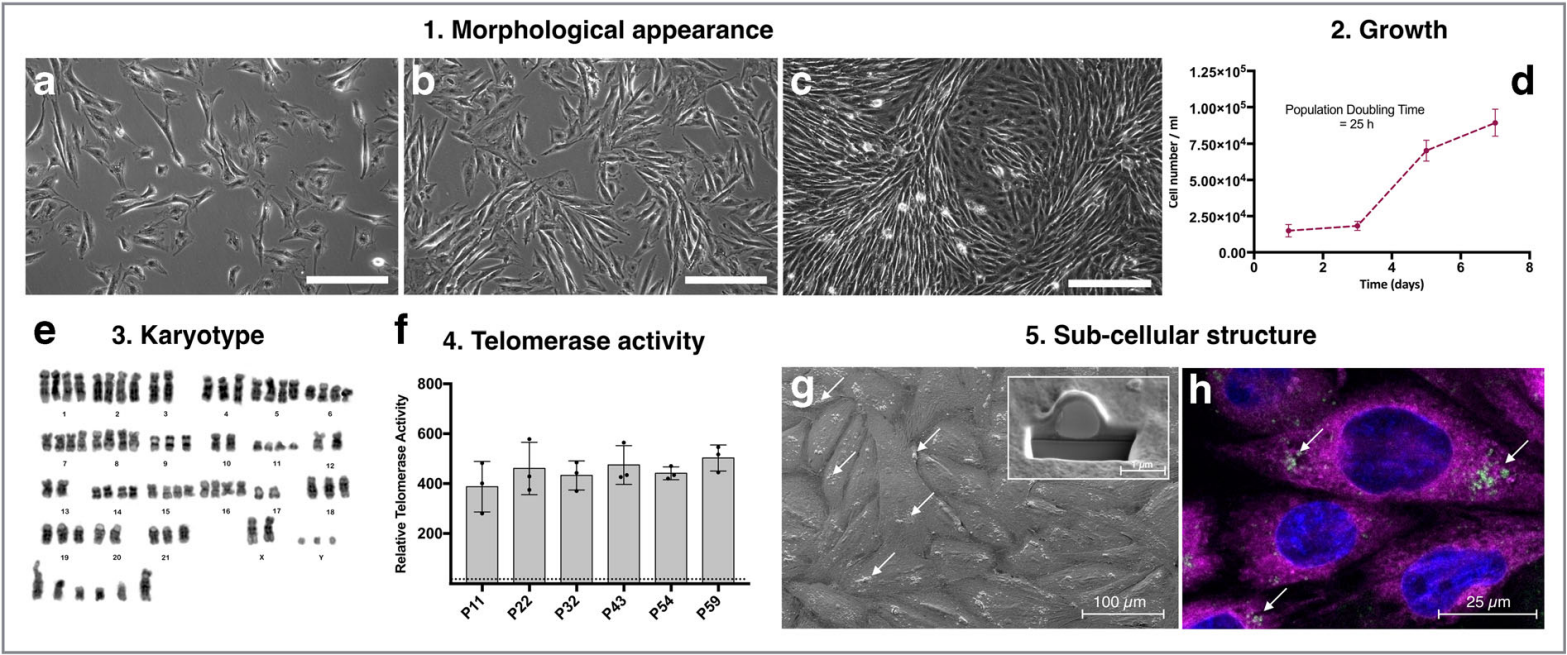
Characterisation of the new strain: HuWa_TERT._ TERT-transfected cells were characterised for five different features. For morphological appearance (1), HuWa_TERT_ cells were cultivated over time and the morphological appearance at different levels of confluency were assessed after 1 day (**a**), 3 days (**b**) and 7 days (**c**). Growth (2) was estimated as cell numbers at 1, 3, 5 and 7 days after plating and the population doubling time calculated during the log phase (**d**). For the karyotype (3), chromosomes of HuWa_TERT_ cells were visualised by G-banding; chromosome pairs of autosomes (1–21), sex chromosomes (X, Y) and unidentified chromosomes are displayed (**e**). The expression of telomerase (4) was measured in HuWa_TERT_ cells at different passages post-transfection (**F**). Data represent the mean of three technical replicates, and the error bars represent the SD for 10,000 cell equivalents. Sub-cellular structures (5) of cells were visualised by  SEM (**G**); the inset shows a cross section of one intracellular lipid body. Arrows indicate lipid bodies as  seen by confocal microscopy (**H**, green and arrows). Other sub-cellular structures visualised are the endoplasmic reticulum (magenta) and cell nucleus (blue)

In contrast, cells transfected with TERT continued to proliferate at an increased rate compared to HuWa_wild-type_. When split 1:3, HuWa_TERT_ formed confluent cell monolayers within 1 week (Fig. 1a–c). Cells exhibited similar attachment efficiencies as HuWa_wild-type_. The population doubling time during the log-growth phase was 25 h (Fig. 1d), compared to 41 h for HuWa_wild-type_. Karyotyping of HuWa_TERT_ (P12, post-transfection) indicated 21 pairs of autosomes and 1 pair of male sex chromosomes, with no obvious structural abnormalities. Cells exhibited chromosome numbers approaching tetraploidy (Fig. 1e).

To test if TERT transfection was successful, HuWa_TERT_ cells were tested for their capacity to express telomerase. All tested passages showed positive telomerase expression, which was also stable over time (Fig. 1f). HuWa_wild-type_ exhibited only baseline activity which was slightly higher than the limit of detection (dotted line in Fig. 1f, close to the X-axis).

SEM and confocal microscopy revealed abundant lipid bodies, between 0.5 and 1 μm in diameter, within the outer cytoplasm (Fig. 1g/h). The endoplasmic reticulum, which is typically quite distinctive in fibroblasts, was very abundant.

### Expression of inflammatory cytokines in immune or chemically challenged HuWa_TERT_

The presence, and concentrations, of the selected inflammatory cytokines was first tested in unstimulated cells. The inflammatory cytokines (IL-6, IL-1β and TNFα), inflammatory chemokine CXCL10 and the general stress marker HSP70 were selected based on the following criteria: expression in fibroblasts, reaction to LPS and response to immunotoxic chemicals (Fig. S5). All three cytokines and HSP70 were detected in unstimulated cell extracts: IL-6 (~ 307 pg/ml), TNFα (~ 6 ng/ml), IL-1β (~ 27 pg/ml) and HSP70 (~ 250 ng/ml). CXCL10 and IFN-γ were not present at detectable levels in HuWa_TERT_ cells. The expression of cytokines was independent from the passage number of HuWa_TERT_; experiments with earlier passages (P 18–25) resulted in comparable results.

**Fig. 2:**
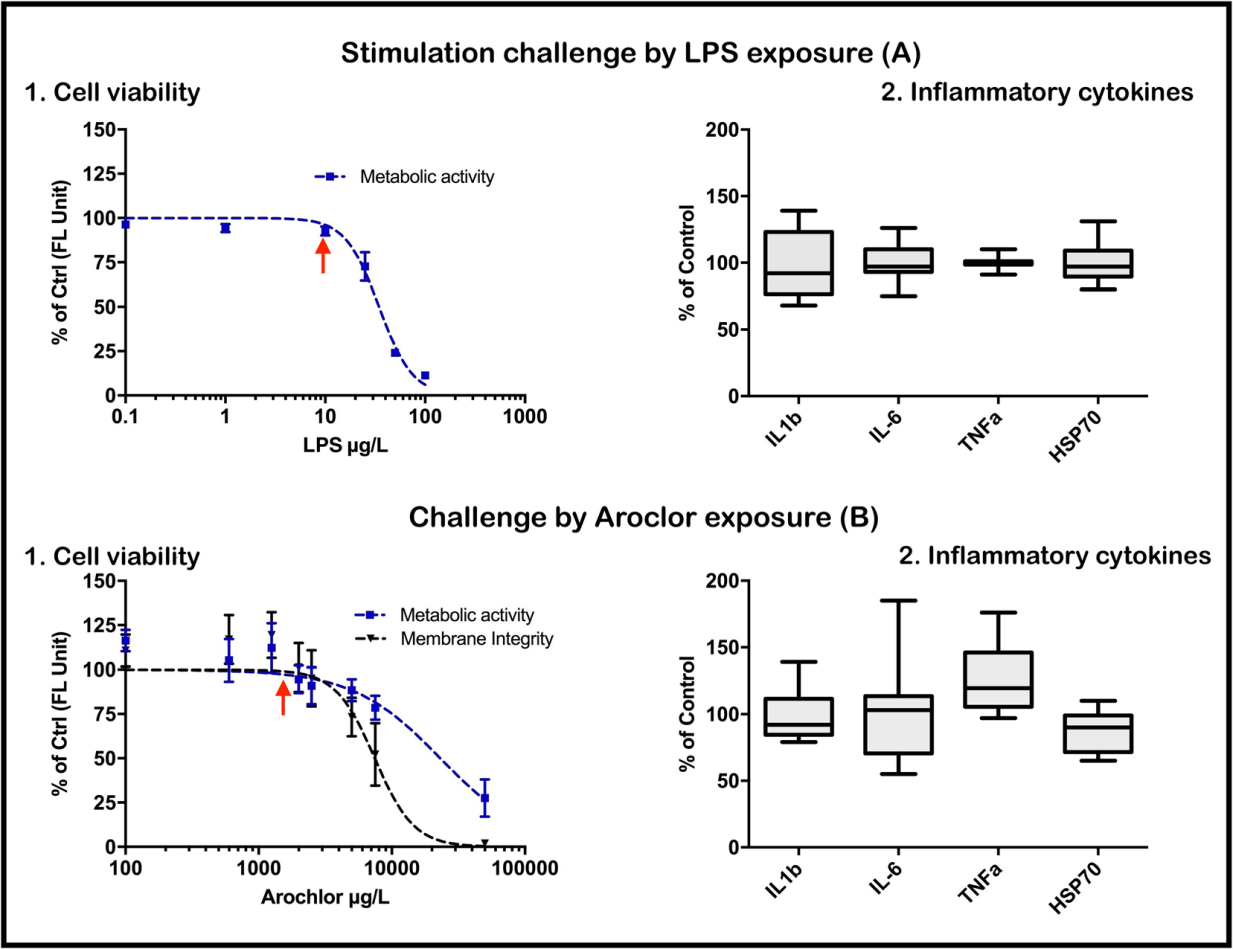
Expression of inflammatory cytokines in HuWa_TERT_ either stimulated with LPS or exposed to Aroclor. For cell viability assessment, HuWa_TERT_ cells were treated with different LPS (A.1) and Aroclor (B.1) concentrations. After 24 h of exposure, metabolic activity using AlamarBlue (LPS and Aroclor) and membrane integrity using CFDA-AM (Aroclor only) were measured. The data in A.1 represents the mean of three technical and, B.1, three biological replicates; the error bars represent SD and are shown as percentage of non-treated cells. The cellular expression of selected inflammatory cytokines (IL-1β, IL-6, TNFα and HSP70) was measured upon treatment with non-cytotoxic concentrations (indicated by red arrow) of LPS (10 μg/ml) (A.2) or Aroclor (1700 μg/l) (B.2) for 24 h. The data is shown as percentage of control. Boxplots indicate median, 5 and 95 percentiles and min/max of three independent biological replicates. No statistical difference was found between single markers and unexposed control using one-way ANOVA and Bonferroni’s post hoc test (*p* < 0.05)

Before the treatment with LPS or Aroclor, non-cytotoxic concentrations (ntC) were ascertained by cell viability assessment. LPS can trigger expression of inflammatory cytokines at various concentrations; however, high concentrations result in cellular toxicity. LPS treatment of 100 and 50 μg/ml clearly reduced metabolic activity of HuWa_TERT_, while concentrations ≤ 10 μg/ml were comparable to the LPS-free control (Fig. 2A1). Exposure to Aroclor also indicated a concentration-dependent cytotoxicity, with a higher reduction of cell viability being observed for membrane integrity (EC_50_ = 7.45 mg/l) compared to metabolic activity (EC_50_ = 23.14 mg/l) (Fig. 2B2). The stimulation challenge with LPS and the chemical challenge upon Aroclor exposure were performed with the non-cytotoxic concentrations of 10 μg/ml (LPS) and 1700 μg/l (Aroclor). For both challenges, the expression of inflammatory cytokines (IL-1β, IL-6, TNFα and HSP70) was not significantly different from levels in unstimulated cells. The cytokines, CXCL10 and IFN-γ, were not detectable in challenged HuWa_TERT_ cells.

## Discussion

This study was designed to develop a consistent in vitro model to ensure long-term preservation of HuWa cell lines, and apply this model to bridge knowledge gaps regarding the immune health of humpback whales, in particular by assessment of sub-cellular responses under immune stimulation and chemical stress.

HuWa_wild-type_ cells were successfully transfected with TERT leading to the establishment of a new strain, HuWa_TERT_. Accordingly, telomerase activity was measureable and stable over at least 59 passages. By contrast, only baseline telomerase activity was found in HuWa_wild-type_ cells. This activity is in line with observed telomerase values in fibroblasts of grey whales (*Eschrichtius robustus*) and fibroblasts of bowhead whales (*Balaena mysticetus*) (Gorbunova and Seluanov 2009). According to population doubling times (PBT), HuWa_TERT_ proliferate more rapidly (25 h) than HuWa_wild-type_ (41 h) (Burkard et al. 2015). The cells display the typical elongated, bipolar and spindle-shaped fibroblast morphology observed in HuWa_wild-type._ Karyotyping revealed that HuWa_TERT_ cells have normal chromosome numbers and structures; however, cells showed tetraploidy which is a commonly observed artefact in cultured cell lines (Leibiger et al. 2013).

The transfection efficiencies of both SV40T plasmids were low, and transient, since very few cells expressed the SV40T protein and, while proliferation rates were initially high, most cells ceased proliferating after just a few passages. SV40T transformation is often associated with non-consistent transfection, genomic variability and changed morphology (Ouellette et al. 2000; Mayne et al. 1986). Indeed, long-term SV40T expression in mammalian cells is relatively rare; it is often reached only together with telomerase activation (Foddis et al. 2002; Li et al. 2008).

By contrast, TERT transfection resulted in clones that divided vigorously, and revealed no distinctive morphological changes or signs of senescence. A non-malignant phenotype, physiological stability (Jiang et al. 1999; Morales et al. 1999) and higher resistance towards DNA damage (Sherr and DePinho 2000) are the major advantages of TERT transfections. Thus far in our studies, HuWa_TERT_ was sub-cultured for 71 passages (12 times prior and 59 times post-transfection), which corresponds to ~ 107 population doublings, assuming 1.5 generations per passage. In un-transformed lung fibroblasts of the bowhead whale, growth arrest was observed after 86 population doublings (Gorbunova and Seluanov 2009). With the long-term preservation, HuWa_TERT_ are a promising, low-cost and easy-to-use resource for fundamental marine wildlife research to advance our current understanding of species-specific physiological responses. Application of cell-based studies can help to identify cellular mechanisms and targets of environmental stressors, such as chemical pollutants, harmful algal toxins and emerging pathogens.

Dermal fibroblasts are immune-active cells and are both targeted by, and actively react to, immune stimulation (Apte 1995; Korn 1997). Fibroblasts can be stimulated in order to express a wide range of different signalling proteins such as cytokines (Van Linthout et al. 2014; Buckley et al. 2001). In this study, the levels of inflammatory cytokines were assessed in immune-stimulated and chemically stressed cells of the new HuWa_TERT_ strain. IL-6, IL-1β, TNFα and HSP70 were constitutively expressed in HuWa_TERT_, while CXCL10 and IFN-γ were not detected with, or without, stimulation/chemical stress. Quantification of the tested cytokines was performed by ELISA which is dependent on successful antibody-target protein reaction. All positively expressed proteins were based on cross-reaction with mammalian antibodies. In general, activity of cytokines and chemokines is relatively well conserved throughout evolution (Kimbrell and Beutler 2001; Secombes et al. 2016; Zou et al. 2016; Zlotnik et al. 2006). The high cross-reactivity of mammalian antibodies with HuWa_TERT_ further alludes to the potential of antibody-based methodologies for immune and protein-based biomarker studies. One possible approach is to apply blubber extracts as an environmentally afflicted tissue and use extended cytokine-based immune screening for a better understanding of specific stress responses, such as local inflammation or wound healing.

Cellular responses upon immune stimulation are complex and differ between different stressors. As such, LPS generally mimics bacterial infections by triggering immune and physiological reactions, in particular the regulation of cytokine levels (Alexander and Rietschel 2001). We investigated the expression of inflammatory markers in HuWa_TERT_ upon treatment with a non-cytotoxic concentration of LPS, which did not result in a significant upregulation of IL-6, IL-1β, TNFα and HSP70. For IL-6 and IL-1β, distinctive upregulation upon LPS stimulation is reported for human fibroblasts (Table S2) suggesting the possibility of a similar response in HuWa_TERT_ cells. However, sensitivity towards LPS is highly species and cell type specific. In human fibroblasts, the ability to regulate cytokines was greatly dependent on the fibroblast type (e.g. wound vs. normal tissue) or differentiation state of fibroblasts (Fibbe et al. 1988; Seelentag et al. 1989; Damme et al. 1989). Further, we tested the effect of different concentrations of LPS, in addition to LPS derived from different species of bacteria. Treatment with different concentrations of LPS (0.1, 1, 10 μg/ml) and LPS from a different species (*Pseudomonas aeruginosa*) or LPS from a different *E. coli* batch (*E. coli* 0111:B4) did not result in upregulation of IL-6 and TNFα expression (Fig. S5). Thus, our findings suggest that HuWa_TERT_ are less sensitive towards LPS compared to human dermal fibroblasts. Besides LPS, other stimulants, such as ovalbumin (a T cell–dependent antigen), concanavalin A (a lymphocyte mitogen) or a virus-based stimulus (poly(I:C)), may be tested to investigate immune regulation in HuWaTERT, comprising future avenues of investigation; one commonly observed pathogen, for example, is the cetacean morbillivirus (CeMV). This virus infects specifically whales, dolphins and porpoise, and several mass mortality events of infected populations have been linked to CeMV epidemics (Van Bressem et al. 2014).

PCBs have been observed to have an immunotoxic impact on marine mammals (Desforges et al. 2016; Mori et al. 2008; Desforges et al. 2017) and were therefore selected as the second applied stressor in this study. Cell viability measurement revealed a lower EC_50_ value for the PCB mixture Aroclor towards membrane integrity, compared to metabolic activity, suggesting perturbation of membranes upon PCB exposure. PCBs are able to cross lipid layers (ATSDR 2000), and, in southern hemisphere humpback whales, PCBs accumulate in the adipose tissue in the range of ΣPCB_32_ < LOD to 720 ng/g_lipid_ (average ~ 20 ng/g_lipid_) (Bengtson Nash et al. 2013; Waugh et al. 2014; Bengtson Nash et al. 2018). If applying a blubber-to-blood diffusion of 0.05% (Cropp et al. 2014) and a blood density of 1.03 g/cm^3^, the estimated blood concentration of PCBs in humpback whales is 9.3 pg/ml. In this study, HuWa_TERT_ were exposed to 1722 ng/ml Aroclor, but no alterations of IL-6, IL-1β, TNFα and HSP70 levels were detectable. No humpback whale study reports the impact of PCBs on these cytokines, and only a few studies have investigated this in marine mammals. In seals with elevated PCB concentrations in their tissue, a correlation between IL-1β levels and increasing PCB concentrations was found (Brown et al. 2014; Routti et al. 2010); on the contrary, in vitro suppressive effects were detected when measuring IL-1β mRNA levels in seal leukocytes (Neale et al. 2005). There is no information about the influence of PCBs on IL-6, TNFα and HSP70 regulation in marine mammals. In humans with elevated PCB concentrations in blood (31–1025 ng/l), no impact was found on IL-6 regulation in human lymphocytes (Daniel et al. 2001). Further, in vitro studies revealed reduced TNFα mRNA levels in murine macrophages upon exposure to PCBs (Santoro et al. 2015), and no change was detected in PCB-exposed human mast cells (Kwon et al. 2002).

Based on the results of this study and the mammalian studies discussed above, there are still significant knowledge gaps regarding the understanding of immune regulation, particularly in combination with exposure to chemicals in marine mammals. The development of the new HuWa_TERT_ strain provides sufficient material for extensive analyses, and is a promising alternative to address these gaps to gain insights in the unique physiological adaptations and molecular responses of humpback whales.

## Conclusion

We demonstrated successful transfer of an exogenous gene into humpback whale fibroblasts. Stable telomerase expression suggests that this cell line can be maintained long term. The positive expression of selected inflammatory cytokines (IL-6, IL-1β, TNFα and HSP70) demonstrates the applicability of this cell line for further immunological and physiological investigations. Overall, it opens mani-fold opportunities to study humpback whale-specific characteristics and cellular response mechanisms.

## Electronic supplementary material

ESM 1 (DOCX 5704 kb)
