# Supplementary material for: Lifetime extension of humpback whale skin fibroblasts and their response to lipopolysaccharide (LPS) and a mixture of polychlorinated biphenyls (Aroclor)
Source: Cell Biol Toxicol. 2019 Jan 10;35(4):387–98. doi: 10.1007/s10565-018-09457-1 (PMC6757015; doi:10.1007/s10565-018-09457-1)
Supplement: Supplementary file 1 — (DOCX 5704 kb) [file 10565_2018_9457_MOESM1_ESM.docx]

## Supplemental Information

Life-time extension of humpback whale skin fibroblasts and their response to lipopolysaccharide (LPS) and a mixture of polychlorinated biphenyls (Arorclor)

Michael Burkard^1,2^, Susan Bengtson Nash^1^, Gessica Gambaro^2^, Deanne Whitworth^3,4^, Kristin Schirmer^2,5,6 #^

*^1^ Griffith University, School of Environment and Science, Brisbane, QLD, Australia*

*^2^ Eawag, Swiss Federal Institute of Technology, Dübendorf, Switzerland*

*^3^ The University of Queensland, School of Veterinary Science, Gatton, QLD, Australia*

*^4^The University of Queensland, Australian Institute for Bioengineering and Nanotechnology, St Lucia, QLD, Australia*

*^5^ ETH Zürich, Institute of Biogechemistry and Pollutant Dynamics, Zürich, Switzerland*

*^6^ EPF Lausanne, School of Architecture, Civil and Environmental Engineering, Lausanne, Switzerland*

*^#^ Corresponding author:*

*Eawag, Swiss Federal Institute of Aquatic Science and Technology*

*Überlandstrasse 133*

*CH-8600*

*Email:* [Kristin.*Schirmer@eawag.ch*](mailto:Kristin.Schirmer@eawag.ch)

*Phone: +41 (0)58 765 5266*

**Table of content**

**Table S1:** Plasmids used in the present study 3

**Table S2:** Literature review and selection of immune markers 4

**Table S3:** Transfected and antibiotic resistant cell colonies 4

**Figure S1:** Antibiotic susceptibility of HuWa_wild-type_ 5

**Figure S2:** Influence of FBS content on cell viability of HuWa_wild-type_  5

**Figure S3:** HuWa_wild-type_ cell morphology upon plasmid transfection 6

**Figure S4:** Expression of SV40T by immunocytochemical staining 6

**Figure S5:** Influence of LPS treatment on expression of IL-6, TNFa and I-TAC 7

**Table S1:** Plasmids used in the present study

|  | **Entrez gene** | **Vector backbone** | **Antibiotic resistance** | **Bacterial resistance** | **Reference** | **DNA ng/μl** |
| --- | --- | --- | --- | --- | --- | --- |
| **pCEP4-hygro-SV40-Tg** | SV40 large T | pCEP4 | Hygromycin | Ampicillin | (Chou et al., 2011) | 1300 |
| **pBABE-puro-hTERT** | TERT | pBABE-puro | Puromycin | Ampicillin | (Counter et al., 1998) | 1107 |
| **pBABE-puro-SV40 LT** | SV40gp6 | pBABE-puro | Puromycin | Ampicillin | (Zhao et al., 2003) | 310 |

**Table S2:** Immune marker selection

**
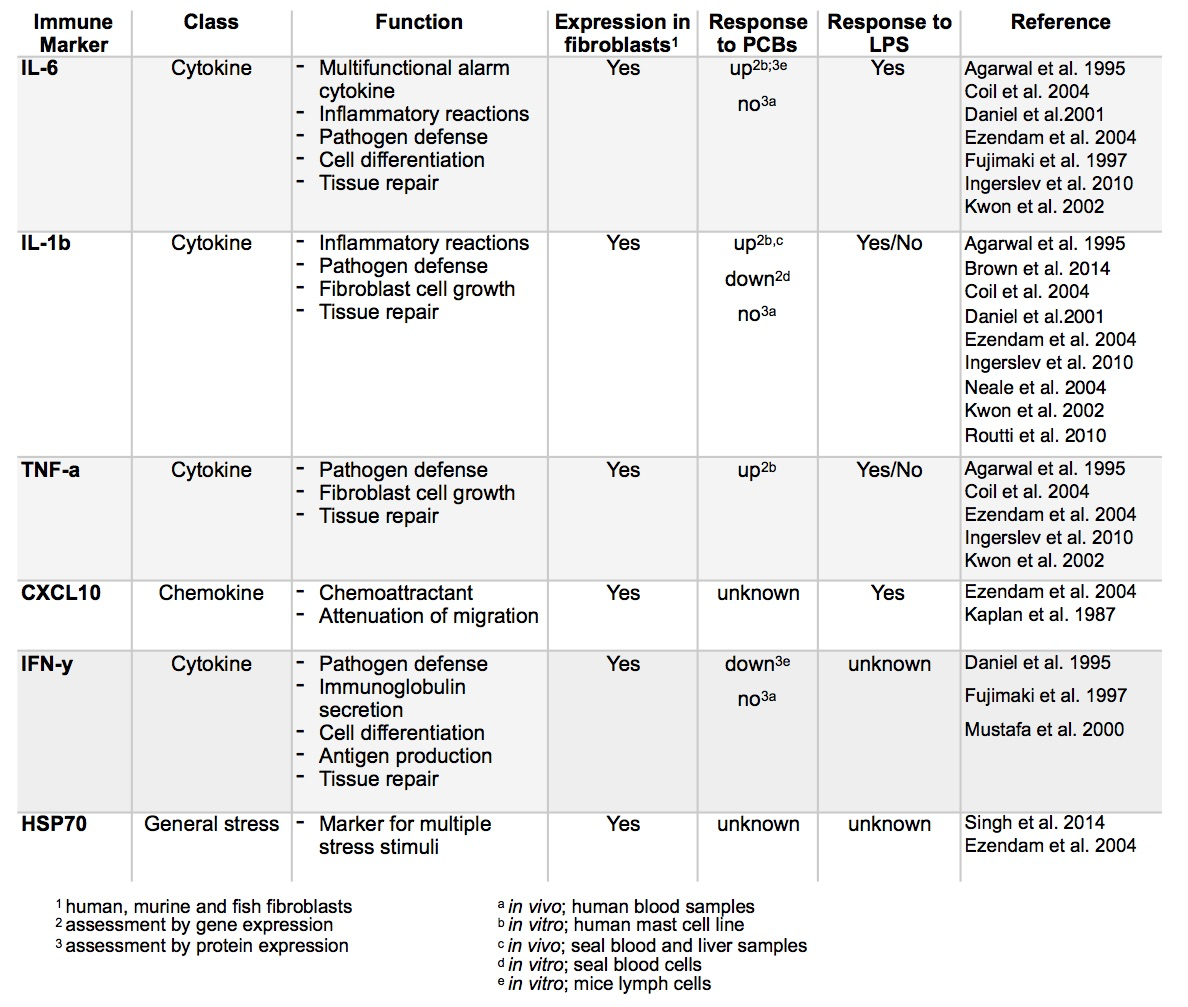
**

**Table S3:** Number of successfully transfected and antibiotic resistant cell colonies

|  |  | **pCEP4-SV40-Tg** | | **pBABE-hTERT** | | **pBABE-SV40 LT** | |
| --- | --- | --- | --- | --- | --- | --- | --- |
|  | Ratio  (DNA/ transfectant) | Fugene | Lipofect. | Fugene | Lipofect. | Fugene | Lipofect. |
| ***Number of colonies*** | *1:1.5*  *1:3*  *1:6* | 4  8  - | 2  4  - | 5  7  4 | 3  4  1 | 1  2  2 | 1  3  - |

**Figure S1:** Antibiotic susceptibility of HuWa_wild-type_

HuWa_wild-type_ were treated with different concentrations of hygromycin and puromycin, and metabolic activity using AlamarBlue was measured after 24 hours of exposure. Data are expressed as percentage of non-treated cells and represent the mean of three replicates and error bars represent SD. The lowest toxic concentrations were chosen for the selection studies and were 100 μg/ml Hygromycin and 25 μg/ml Puromycin.

**Figure S2:** Influence of FBS content on cell viability of HuWa_wild-type_

HuWawild-type were incubated with different concentrations of DMEM/F12 medium containing different FBS percentages. The metabolic activity was measured using AlamarBlue after 24 hours of incubation. Data is expressed as percentage of full medium (10% FBS) and represents the mean of three replicates and error bars represent SD.


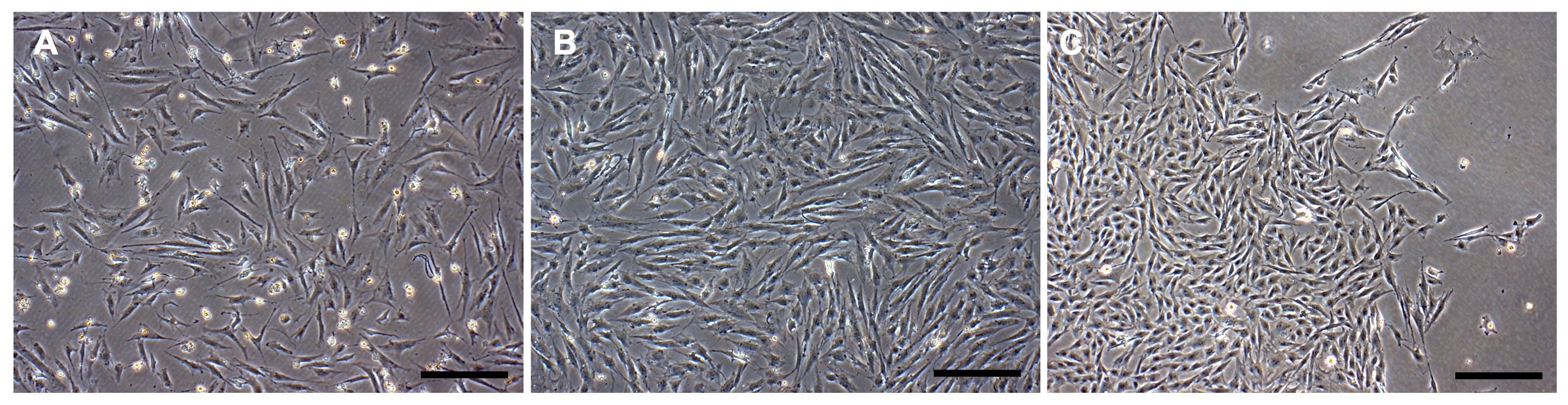


Figure S3: Cell morphology upon transfection of HuWa_wild-type_

HuWa_wild-type_ were transfected with Lipofectamine (A) and Fugene (B) at 1:3 DNA / transfectant ratio, and 24 h post transfection morphology was visualized. Post antibiotic selection, first resistant cell colonies appeared after several days (C) (1:3, Lipofectamine). Scale bar indicates 300 μm.


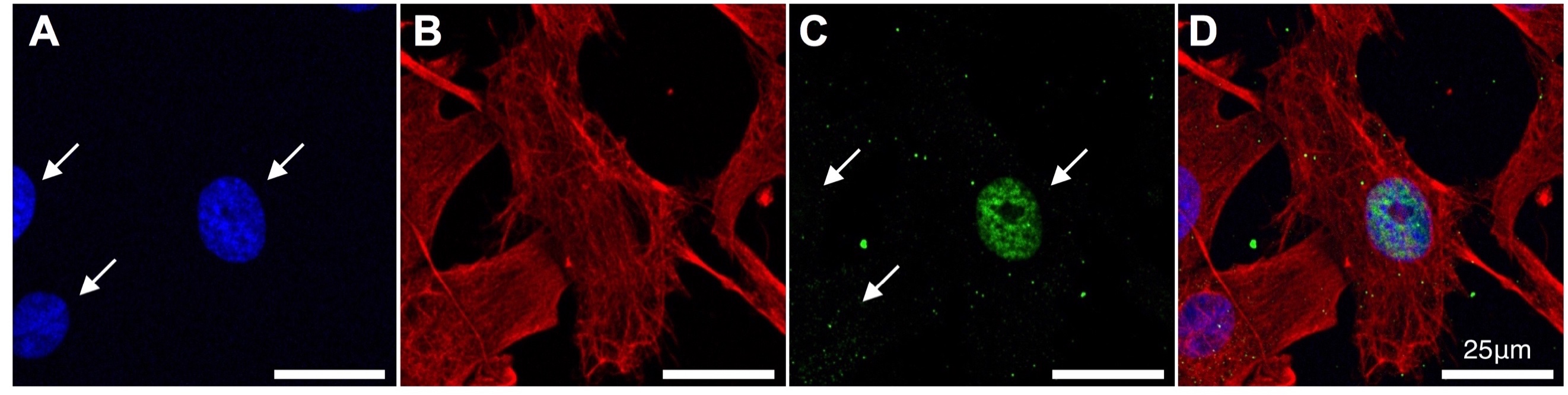


**Figure S4:** Expression of SV40T as assessed by immunocytochemical staining

HuWa_SV40T_ were stained with anti SV40T antibody eight passages post transfection with pBABE-puro-SV40 LT. Cell nuclei are shown in blue (A), cytoskeleton in red (B), SV40T in green (C). The overlay is shown in (D). White arrows in A and C indicate positive expression of SV40T in one out of three cell nuclei.


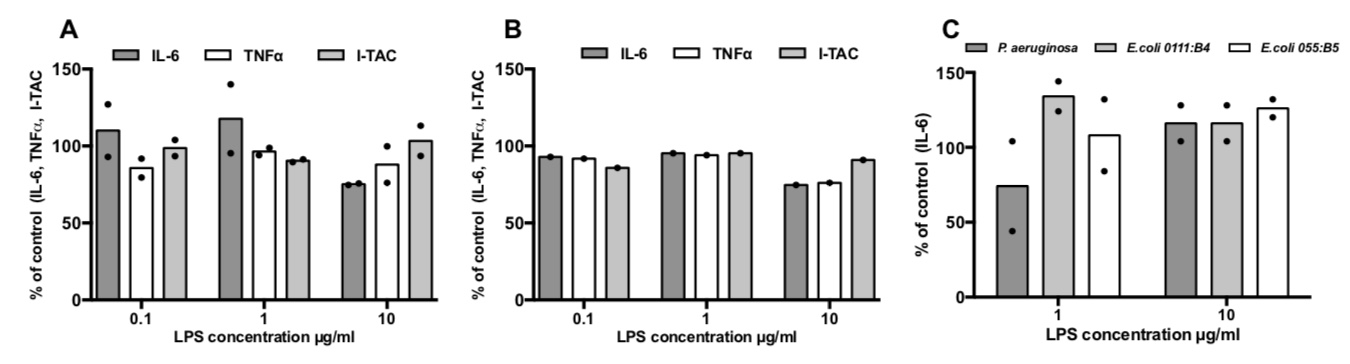

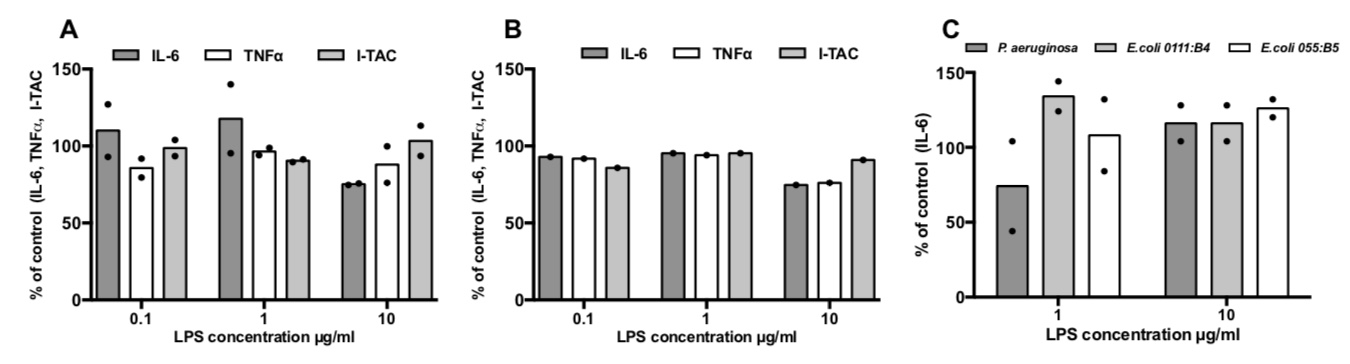


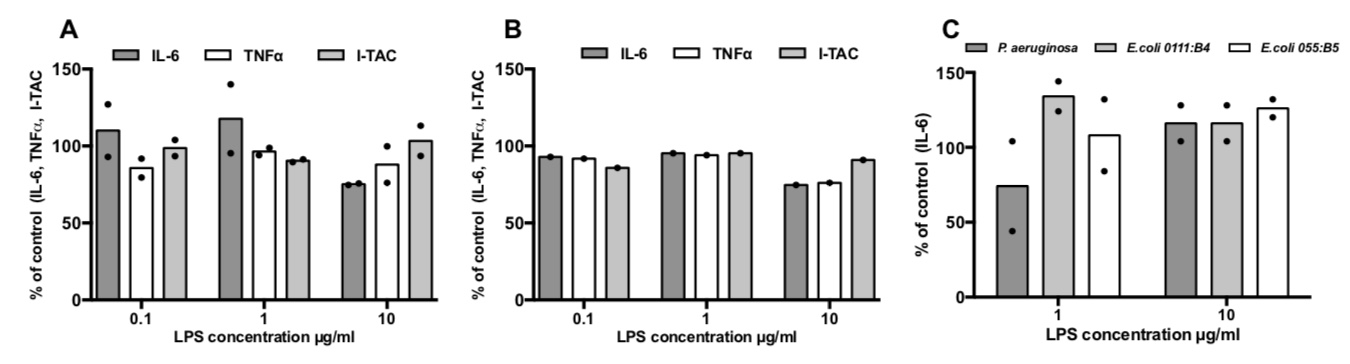


**Figure S5:** Influence of LPS treatment on expression of IL-6, TNFa and I-TAC

A HuWa_TERT_ were treated with different LPS concentrations for 24 hours, and cellular expression of IL-6, TNFa and I-TAC, shown as percentage of control. Bars indicate the mean and circles represent the replicates.

B HuWa_TERT_ were treated with different LPS concentrations for 48 hours and cellular expression of IL-6, TNFa and I-TAC, shown as percentage of control. Bars and circles represent a single measurement.

C HuWa_TERT_ were treated with LPS from different species or strains (*P.aerug; E.coli.0111:B4* and *E.coli. 055:B5*) for 24 hours and cellular expression of IL-6, shown as percentage of control. Bars indicate the mean and circles represent the replicates.

## References

AGARWAL, S., BARAN, C., PIESCO, N., QUINTERO, J., LANGKAMP, H., JOHNS, L. & CHANDRA, C. 1995. Synthesis of proinflammatory cytokines by human gingival fibroblasts in response to lipopolysaccharides and interleukin‐1β. *Journal of periodontal research,* 30**,** 382-389.

BROWN, T. M., ROSS, P. S., REIMER, K. J., VELDHOEN, N., DANGERFIELD, N. J., FISK, A. T. & HELBING, C. C. 2014. PCB Related Effects Thresholds As Derived through Gene Transcript Profiles in Locally Contaminated Ringed Seals (Pusa hispida). *Environmental Science & Technology,* 48**,** 12952-12961.

CHOU, B. K., MALI, P., HUANG, X., YE, Z., DOWEY, S. N., RESAR, L. M., ZOU, C., ZHANG, Y. A., TONG, J. & CHENG, L. 2011. Efficient human iPS cell derivation by a non-integrating plasmid from blood cells with unique epigenetic and gene expression signatures. *Cell Res,* 21**,** 518-29.

COIL, J., TAM, E. & WATERFIELD, J. D. 2004. Proinflammatory cytokine profiles in pulp fibroblasts stimulated with lipopolysaccharide and methyl mercaptan. *Journal of endodontics,* 30**,** 88-91.

COUNTER, C. M., HAHN, W. C., WEI, W., CADDLE, S. D., BEIJERSBERGEN, R. L., LANSDORP, P. M., SEDIVY, J. M. & WEINBERG, R. A. 1998. Dissociation among in vitro telomerase activity, telomere maintenance, and cellular immortalization. *Proceedings of the National Academy of Sciences,* 95**,** 14723-14728.

DANIEL, V., HUBER, W., BAUER, K., SUESAL, C., CONRADT, C. & OPELZ, G. 2001. Associations of blood levels of PCB, HCHS, and HCB with numbers of lymphocyte subpopulations, in vitro lymphocyte response, plasma cytokine levels, and immunoglobulin autoantibodies. *Environmental Health Perspectives,* 109**,** 173-178.

EZENDAM, J., STAEDTLER, F., PENNINGS, J., VANDEBRIEL, R. J., PIETERS, R., HARLEMAN, J. H. & VOS, J. G. 2004. Toxicogenomics of subchronic hexachlorobenzene exposure in Brown Norway rats. *Environ Health Perspect,* 112**,** 782-91.

FUJIMAKI, H., SHIRAISHI, F., AOKI, Y. & SANEYOSHI, K. 1997. Modulated cytokine production from cervical lymph node cells treated with B[a]P and PCB. *Chemosphere,* 34**,** 1487-93.

INGERSLEV, H. C., OSSUM, C. G., LINDENSTROM, T. & NIELSEN, M. E. 2010. Fibroblasts express immune relevant genes and are important sentinel cells during tissue damage in rainbow trout (Oncorhynchus mykiss). *PLoS One,* 5**,** e9304.

KWON, O., LEE, E., MOON, T. C., JUNG, H., LIN, C. X., NAM, K.-S., BAEK, S. H., MIN, H.-K. & CHANG, H. W. 2002. Expression of Cyclooxygenase-2 and Pro-inflammatory Cytokines Induced by 2,2&prime;,4,4&prime;,5,5&prime;-Hexachlorobiphenyl (PCB 153) in Human Mast Cells Requires NF-&kappa;B Activation. *Biological and Pharmaceutical Bulletin,* 25**,** 1165-1168.

NEALE, J. C. C., KENNY, T. P., TJEERDEMA, R. S. & GERSHWIN, M. E. 2005. PAH- and PCB-induced Alterations of Protein Tyrosine Kinase and Cytokine Gene Transcription in Harbor Seal (Phoca Vitulina) PBMC. *Clinical and Developmental Immunology,* 12**,** 91-97.

ROUTTI, H., ARUKWE, A., JENSSEN, B. M., LETCHER, R. J., NYMAN, M., BACKMAN, C. & GABRIELSEN, G. W. 2010. Comparative endocrine disruptive effects of contaminants in ringed seals (Phoca hispida) from Svalbard and the Baltic Sea. *Comp Biochem Physiol C Toxicol Pharmacol,* 152**,** 306-12.

SINGH, A. K., UPADHYAY, R. C., MALAKAR, D., KUMAR, S. & SINGH, S. V. 2014. Effect of thermal stress on HSP70 expression in dermal fibroblast of zebu (Tharparkar) and crossbred (Karan-Fries) cattle. *J Therm Biol,* 43**,** 46-53.

ZHAO, J. J., GJOERUP, O. V., SUBRAMANIAN, R. R., CHENG, Y., CHEN, W., ROBERTS, T. M. & HAHN, W. C. 2003. Human mammary epithelial cell transformation through the activation of phosphatidylinositol 3-kinase. *Cancer Cell,* 3**,** 483-95.
